# Increasing Capacity to Detect Clusters of Rapid HIV Transmission in Varied Populations—United States

**DOI:** 10.3390/v13040577

**Published:** 2021-03-30

**Authors:** Alexandra M. Oster, Nivedha Panneer, Sheryl B. Lyss, R. Paul McClung, Meg Watson, Neeraja Saduvala, M. Cheryl Bañez Ocfemia, Laurie Linley, William M. Switzer, Joel O. Wertheim, Ellsworth Campbell, Angela L. Hernandez, Anne Marie France

**Affiliations:** 1 Centers for Disease Control and Prevention, Atlanta, GA 30329, USA; npanneer@cdc.gov (N.P.); Slyss@cdc.gov (S.B.L.); rmcclung@cdc.gov (R.P.M.); eze5@cdc.gov (M.W.); cocfemia@cdc.gov (M.C.B.O.); llinley@cdc.gov (L.L.); bis3@cdc.gov (W.M.S.); ykk7@cdc.gov (E.C.); ahernandez@cdc.gov (A.L.H.); afrance@cdc.gov (A.M.F.); 2 U.S. Public Health Service, Atlanta, GA 30329, USA; 3 ICF International, Atlanta, GA 30329, USA; nsaduvala@cdc.gov; 4 Department of Medicine, University of California San Diego, San Diego, CA 92093, USA; jwertheim@ucsd.edu

**Keywords:** HIV-1, cluster analysis, epidemiology, public health, substance abuse, intravenous, sexual and gender minorities

## Abstract

Molecular cluster detection analyzes HIV sequences to identify rapid HIV transmission and inform public health responses. We describe changes in the capability to detect molecular clusters and in geographic variation in transmission dynamics. We examined the reporting completeness of HIV-1 polymerase sequences in quarterly National HIV Surveillance System datasets from December 2015 to December 2019. Priority clusters were identified quarterly. To understand populations recently affected by rapid transmission, we described the transmission risk and race/ethnicity of people in clusters first detected in 2018–2019. During December 2015 to December 2019, national sequence completeness increased from 26% to 45%. Of the 1212 people in the 136 clusters first detected in 2018–2019, 69% were men who have sex with men (MSM) and 11% were people who inject drugs (PWID). State-by-state analysis showed substantial variation in transmission risk and racial/ethnic groups in clusters of rapid transmission. HIV sequence reporting has increased nationwide. Molecular cluster analysis identifies rapid transmission in varied populations and identifies emerging patterns of rapid transmission in specific population groups, such as PWID, who, in 2015–2016, comprised only 1% of people in such molecular clusters. These data can guide efforts to focus, tailor, and scale up prevention and care services for these populations.

## 1. Introduction

Responding to HIV clusters and outbreaks is one of the four pillars of the U.S. Ending the HIV Epidemic (EHE) initiative, which aims to reduce new HIV infections by 90% by 2030. This initiative is initially focusing on 48 counties; Washington, D.C.; San Juan, Puerto Rico, and seven states with substantial rural burden (i.e., EHE jurisdictions) [1].

Analyzing HIV nucleotide sequences is one method to identify clusters of rapid transmission for public health responses [2,3,4]. Analysis of HIV sequences reported to the National HIV Surveillance System (NHSS) to identify molecular clusters began with data submitted through December 2015. At that time, 22 state/territorial and five local health departments were funded by the Centers for Disease Prevention and Control (CDC) to collect sequences through the laboratory reporting of sequences generated by drug resistance testing as a routine part of clinical care. In 2018, requirements to collect HIV sequences expanded to all CDC-funded health departments (i.e., 50 state, two territorial, and seven local health departments) [5]. 

Analysis of data from 2015–2016 on clusters of rapid transmission identified by CDC analysis of HIV nucleotide sequences showed that most people in these clusters were gay, bisexual, and other men who have sex with men (MSM), and only 1% were people who inject drugs (PWID) [6]. However, transmission dynamics can change over time and can vary geographically.

We aimed to describe temporal changes in national sequence completeness, a critical measure of molecular cluster detection capability [7], in the United States. We also examined geographic variation in the transmission category and race/ethnicity of people in clusters of rapid transmission.

## 2. Materials and Methods

To understand changes in a key component of molecular cluster detection capability, for each quarterly NHSS data set from December 2015 (start of molecular cluster detection) to December 2019, we calculated HIV-1 polymerase (*pol*) sequence reporting completeness (i.e., the percentage of all diagnoses in the past 3 years with an HIV sequence available). Sequences that were <500 nucleotides or of poor quality were excluded. These HIV-1 *pol* sequences were generated through HIV drug resistance testing conducted at commercial, private, and public laboratories as part of standard HIV care. Sequences were reported to state and local health departments and entered into local surveillance systems. Data were then submitted to the CDC, without personal identifiers, as part of routine HIV surveillance reporting.

For each quarterly dataset, we then identified clusters of rapid transmission among people with HIV diagnosed in the past 3 years using HIV TRAnsmission Cluster Engine (HIV-TRACE) [8]. As previously described, we analyzed a 1497-nucleotide segment of the protease and reverse transcriptase genes, used a pairwise genetic distance threshold of 0.5%, and identified priority clusters as those with ≥5 diagnoses in the preceding 12 months [3]. Beginning with the June 2016 dataset, we included all available sequences for each person.

After determining the total number of priority clusters identified during December 2015 to December 2019, we then focused further analyses on clusters first detected in 2018–2019, after expansion of sequence reporting. For people in these clusters, we described the transmission category and race/ethnicity. For this analysis, the transmission category was not imputed. Mutually exclusive categories were: people with HIV attributable to male-to-male sexual contact (i.e., MSM); injection drug use (i.e., PWID); male-to-male sexual contact and injection drug use (i.e., MSM who inject drugs); heterosexual contact (i.e., heterosexual people); other (including no identified risk or perinatal). Race/ethnicity was grouped into mutually exclusive categories: Black/African American (hereafter referred to as Black), Hispanic/Latino, White, and other (includes American Indian/Alaska Native, Asian, Native Hawaiian or Other Pacific Islander, and people of multiple races).

Next, for the 19 states that had at least 20 total people included in priority clusters first detected in 2018–2019, we stratified results related to the transmission category and race/ethnicity by state. To determine whether variation in characteristics of people in clusters was simply a reflection of differences in populations affected by HIV in each state or of sequence completeness, we also compared the characteristics of people in clusters to those of people in that state with HIV diagnoses during 2018–2019 and assessed sequence completeness for each subgroup. To facilitate comparisons, the transmission category was not imputed for either group. Individual states are not identified, in accordance with data re-release agreements between the CDC and health departments.

## 3. Results

**Figure 1:**
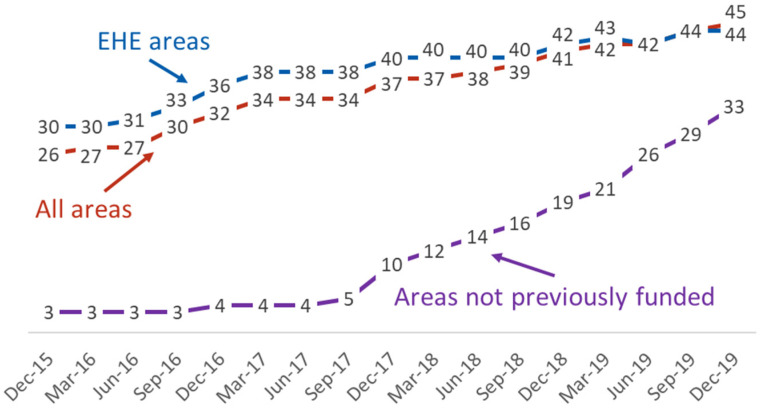
Sequence completeness (percentage of diagnoses in the past 3 years with an HIV sequence available), by quarterly dataset, in Ending the HIV Epidemic (EHE) priority areas, all areas, and areas not previously funded to collect sequence data before 2018.

Sequence reporting completeness (for diagnoses in the past 3 years) in quarterly datasets from December 2015 to December 2019 increased nationwide (from 26% to 45%), in initial EHE jurisdictions (from 30% to 44%), and in areas not previously funded to collect sequences (from 3% to 33%) (Figure 1). For the December 2019 data set, this meant that 49,777 (45%) of the 111,128 diagnoses that occurred during 2017–2019 and were reported through December 2019 had an analyzable sequence.

During December 2015 to December 2019, 242 priority clusters were detected. Of these, 136 clusters (56%) were first detected in 2018–2019, after the expansion of sequence reporting. These clusters comprised a total of 1212 people at the time of detection. The median cluster size at the time of detection was eight (range: 5–24). Of the 1212 people in these 136 clusters, 841 (69%) were MSM (i.e., had HIV attributable to male-to-male sexual contact), 137 (11%) were PWID (i.e., had HIV attributable to injection drug use), 70 (6%) had HIV attributable to heterosexual contact, 56 (5%) were MSM who inject drugs (i.e., had HIV attributable to male-to-male sexual contact and injection drug use), and 108 (9%) had no identified or other risk. By race/ethnicity, 441 (36%) of people in priority clusters were White, 371 (31%) were Black, 344 (28%) were Hispanic/Latino, and 56 (5%) were other races. Overall, 56% of people in priority clusters resided in EHE jurisdictions at diagnosis; EHE jurisdictions represented 55% of all HIV diagnoses occurring during 2018–2019 and reported by December 2019.

State-by-state analysis was limited to states in which at least 20 cluster members resided at HIV diagnosis. These 19 states, which included 1113 (92%) of the 1212 people in clusters first detected in 2018–2019, had a median of 1781 diagnoses during 2018–2019 (range: 604–8927) and median sequence completeness of 52% (range: 34–67%). The states were in all four U.S. Census regions (Northeast: three; Midwest: two; South: 10; West: four). Of these 19 states, 17 included areas funded to collect sequences before 2018, and 13 contained EHE jurisdictions.

**Figure 2:**
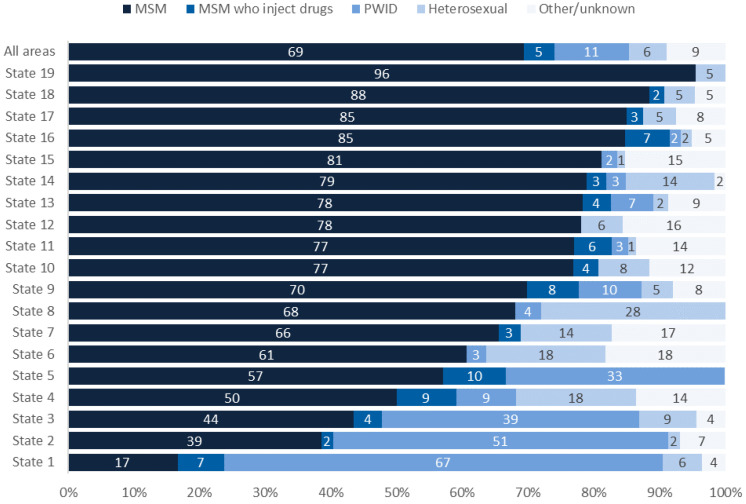
Transmission category of people in clusters of rapid transmission first detected during 2018–2019, overall and in selected states (those with at least 20 cluster members). MSM: men who have sex with men; PWID: people who inject drugs.

This state-by-state analysis showed tremendous variation in the transmission category and race/ethnicity of people in clusters of rapid transmission. The percentage of MSM in clusters ranged from 17% in state 1 to 96% in state 19 (Figure 2). The percentage of PWID ranged from 0% in multiple states to 67% in state 1, and the percentage of heterosexual people ranged from 1% in multiple states to 28% in state 8.

The distribution of the transmission category of people in clusters was not simply a reflection of populations with HIV diagnoses in each state nor of variations in sequence completeness (Table 1). For example, in state 14, MSM represented a larger proportion of people in clusters than people with HIV diagnoses during 2018–2019 (79% vs. 55%), and heterosexual people represented a smaller proportion (14% vs. 29%); sequence completeness was similar for MSM (45%) and heterosexual people (48%). In state 8, MSM represented a larger proportion of people in clusters than people with HIV who were diagnosed during 2018–2019 (68% vs. 43%), and sequence completeness showed minor differences for MSM (71%) and all people with diagnoses (65%). In state 1, MSM represented a lower proportion of people in clusters than people with HIV diagnoses (17% vs. 39%), and PWID represented a larger proportion (67% vs. 12%); sequence completeness varied minimally (73% for PWID vs. 63% for all people with diagnoses). 

**Figure 3:**
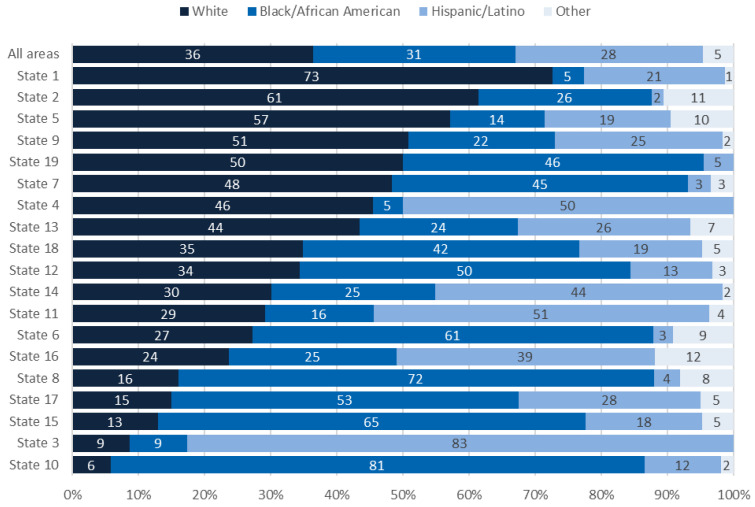
Race/ethnicity of people in clusters of rapid transmission first detected during 2018–2019, overall and in selected states (those with at least 20 cluster members).

Similarly, the race/ethnicity of people in clusters varied among states (Figure 3). The percentage of people in clusters who were White ranged from 6% in state 10 to 73% in state 1. The percentage of Black people ranged from 5% in states 1 and 4 to 81% in state 10, and the percentage of Hispanic/Latino people ranged from 2% in state 2 to 83% in state 3.

As with the transmission category, the variation in race/ethnicity by state was not simply a reflection of the different populations with HIV diagnoses during that time period in each state (Table 1). For example, in state 15, Black people represented 65% of the people in clusters but only 36% of people with HIV diagnoses, whereas Hispanic/Latino people represented 18% of the people in clusters and 41% of people with HIV diagnoses. Sequence completeness was similar for Black people (40%) and Hispanic/Latino people (42%). Meanwhile, in state 1, White people represented 73% of the people in clusters compared with 34% of people with HIV diagnoses, and sequence completeness was similar for White people (67%) and all people (63%).

## 4. Discussion

From December 2015 to December 2019, HIV sequence reporting completeness increased nationwide, including in EHE jurisdictions and especially in areas not previously funded to collect sequence data. Together with the expanded utilization of tools allowing local cluster analysis [9,10], these data clearly demonstrate an increased capacity to detect clusters of rapid transmission [7].

This analysis also demonstrates that molecular cluster analysis can identify rapid transmission in varied populations, which may change over time. For example, molecular cluster analysis has detected increased rapid transmission among PWID in recent years [11] (11% in 2018–2019 compared with 1% in 2015–2016) [6]. Moreover, such analysis can highlight rapid transmission even when the groups among which transmission is occurring differ from the groups among which new diagnoses most commonly occur, as seen in several states in this analysis. 

It is critical to note that detected molecular clusters likely represent a small proportion of the true transmission networks, given that people can only be found to be in a molecular cluster if they have received a diagnosis of HIV, entered care, and had a drug resistance test conducted and the resulting sequence was reported to the health department. For example, a molecular cluster of 27 people in San Antonio, Texas was a signal of a much larger network that included at least 88 other people with HIV and many other people without HIV [12]. Considering the entire network being affected by rapid transmission is important for successful cluster response, particularly because those in a molecular cluster are more likely to be in care, whereas others in the network might be more likely to be in need of linkage to testing, care, and prevention services. Moreover, the fact that nearly half of people in priority clusters resided in areas that are not EHE jurisdictions indicates a need to ensure that cluster and outbreak response capacity is available in all U.S. jurisdictions.

Additionally, wide variations in the transmission risk and race/ethnicity of cluster members from different states indicate that prevention and response approaches need to be tailored locally for affected communities. The presence of a cluster of rapid transmission indicates that existing prevention and care services are not adequately reaching people at highest risk of HIV. Often, these people experience marginalization because of multiple and overlapping factors, such as sex, gender identity, racism, language, drug use, and economic disadvantage. For services to be utilized, they must be accessible and welcoming to the populations who need them most. 

Understanding not only the demographic and risk characteristics of people affected by rapid transmission, but also the relevant social and structural context, are necessary so that efforts can be made to improve services, reduce transmission, and improve health outcomes for people living with HIV. For example, responses to clusters meeting molecular priority criteria in Massachusetts, San Antonio, Seattle, and Northern Kentucky and Ohio, have identified specific barriers facing networks experiencing rapid transmission and addressed those barriers to improve services such as testing, HIV care, pre-exposure prophylaxis, and syringe services programs [12,13,14,15]. Additional implementation science and demonstration projects can help health departments and the CDC to gain further experience regarding optimal approaches to such response efforts.

Our work is subject to some limitations. First, our priority cluster definition for this analysis used a threshold of five diagnoses in the preceding 12 months. However, clusters with as few as three diagnoses in the preceding 12 months have equally high growth and transmission rates [3,16]. The CDC currently applies the lower threshold to areas with lower numbers of HIV diagnoses, and some health departments in higher burden areas that have capacity to respond earlier also use lower thresholds. Additionally, maximizing sequence completeness is important for maximizing the detection of clusters [7]. Our analyses showed that the variability in the transmission and race/ethnicity categories for people in priority clusters was not due to sequence completeness. However, clusters occurring in states with low sequence completeness would have been less likely to be detected and included in this analysis.

## 5. Conclusions

In summary, molecular cluster detection offers a focused, local approach to identify populations experiencing rapid transmission and to tailor efforts to scale up services for these populations. This is a critical addition to the data-driven decision-making about routine prevention and care efforts that is already incorporated into HIV programs. These results demonstrate that use of molecular cluster detection provides great potential for guiding public health responses to clusters and outbreaks.

## Figures and Tables

**Table 1:** Transmission category and race/ethnicity of people with HIV diagnosed in 2018–2019 and people in molecular HIV clusters first detected in 2018–2019, by state. MSM: men who have sex with men; PWID: people who inject drugs. Only includes states with at least 20 cluster members.

		Transmission Category					Race/Ethnicity			
		MSM	MSM Who Inject Drugs	PWID	Heterosexual	Other	White	Black	Hispanic/Latino	Other
		%	%	%	%	%	%	%	%	%
State 1	All diagnoses	39	3	12	8	38	34	31	28	6
	Cluster members	17	7	67	6	4	73	5	21	1
State 2	All diagnoses	49	4	11	8	29	43	47	6	4
	Cluster members	39	2	51	2	7	61	26	2	11
State 3	All diagnoses	50	6	9	7	28	43	26	21	10
	Cluster members	44	4	39	9	4	83	0	9	9
State 4	All diagnoses	58	8	5	10	18	44	15	36	4
	Cluster members	50	9	9	18	14	46	5	50	0
State 5	All diagnoses	52	6	14	6	23	62	28	6	4
	Cluster members	57	10	33	0	0	57	14	19	10
State 6	All diagnoses	46	1	2	10	42	22	73	2	3
	Cluster members	61	0	3	18	18	27	61	3	9
State 7	All diagnoses	42	2	4	19	33	22	68	8	2
	Cluster members	66	3	0	14	17	48	45	3	3
State 8	All diagnoses	43	1	4	27	26	12	74	9	5
	Cluster members	68	0	4	28	0	16	72	4	8
State 9	All diagnoses	49	3	10	14	24	31	47	18	4
	Cluster members	70	8	10	5	8	51	22	25	2
State 10	All diagnoses	49	1	2	14	35	16	72	9	3
	Cluster members	77	4	0	8	12	6	81	12	2
State 11	All diagnoses	62	4	5	6	24	25	18	49	9
	Cluster members	77	6	3	1	14	29	16	51	4
State 12	All diagnoses	48	2	2	9	40	25	65	7	3
	Cluster members	78	0	0	6	16	34	50	13	3
State 13	All diagnoses	63	5	6	7	20	32	18	41	9
	Cluster members	78	4	7	2	9	44	24	26	7
State 14	All diagnoses	55	2	4	29	11	24	39	35	2
	Cluster members	79	3	3	14	2	30	25	44	2
State 15	All diagnoses	54	3	3	7	34	20	36	41	5
	Cluster members	81	0	2	1	15	13	65	18	5
State 16	All diagnoses	55	2	3	10	30	15	42	36	8
	Cluster members	85	7	2	2	5	24	25	39	12
State 17	All diagnoses	53	3	2	13	30	23	61	11	4
	Cluster members	85	3	0	5	8	15	53	28	5
State 18	All diagnoses	57	2	2	12	27	21	51	24	4
	Cluster members	88	2	0	5	5	35	42	19	5
State 19	All diagnoses	49	3	5	7	37	33	56	8	3
	Cluster members	96	0	0	5	0	50	46	5	0

## Data Availability

The data used in this analysis were collected by state health departments and shared with the CDC as part of the National HIV Surveillance System. HIV surveillance data reported to CDC are protected under a federal Assurance of Confidentiality set forth in Section 308(d) of the Public Health Service Act that limits the release of the data. These data can be released only for public health purposes in accordance with the policies for data release established by the Council of State and Territorial Epidemiologists and data re-release agreements between the CDC and health departments. As these agreements allow for the release of HIV surveillance data in aggregate form only, the CDC is unable to provide primary individual level data for external analyses.
